# Inhibition of Sarco-Endoplasmic Reticulum Ca^2+^ ATPase Extends the Lifespan in *C. elegans* Worms

**DOI:** 10.3389/fphar.2018.00669

**Published:** 2018-06-25

**Authors:** Paloma García-Casas, Jessica Arias-del-Val, Pilar Alvarez-Illera, Rosalba I. Fonteriz, Mayte Montero, Javier Alvarez

**Affiliations:** Department of Biochemistry and Molecular Biology and Physiology, Institute of Biology and Molecular Genetics (IBGM), Faculty of Medicine, University of Valladolid – CSIC, Valladolid, Spain

**Keywords:** *C. elegans*, lifespan, Ca^2+^ signaling, SERCA, thapsigargin, aging, calcium, endoplasmic reticulum

## Abstract

The sarco-endoplasmic reticulum Ca^2+^-ATPase (SERCA) refills the endoplasmic reticulum (ER) with Ca^2+^ up to the millimolar range and is therefore the main controller of the ER [Ca^2+^] level ([Ca^2+^]_ER_), which has a key role in the modulation of cytosolic Ca^2+^ signaling and ER-mitochondria Ca^2+^ transfer. Given that both cytosolic and mitochondrial Ca^2+^ dynamics strongly interplay with energy metabolism and nutrient-sensitive pathways, both of them involved in the aging process, we have studied the effect of SERCA inhibitors on lifespan in *C. elegans*. We have used thapsigargin and 2,5-Di-tert-butylhydroquinone (2,5-BHQ) as SERCA inhibitors, and the inactive analog 2,6-Di-tert-butylhydroquinone (2,6-BHQ) as a control for 2,5-BHQ. Every drug was administered to the worms either directly in the agar or via an inclusion compound with γ-cyclodextrin. The results show that 2,6-BHQ produced a small but significant increase in survival, perhaps because of its antioxidant properties. However, 2,5-BHQ produced in all the conditions a much higher increase in lifespan, and the potent and specific SERCA inhibitor thapsigargin also extended the lifespan. The effects of 2,5-BHQ and thapsigargin had a bell-shaped concentration dependence, with a maximum effect at a certain dose and smaller or even toxic effects at higher concentrations. Our data show therefore that submaximal inhibition of SERCA pumps has a pro-longevity effect, suggesting that Ca^2+^ signaling plays an important role in the aging process and that it could be a promising novel target pathway to act on aging.

## Introduction

The molecular mechanisms responsible for aging are not yet fully understood. In recent years, evidence has accumulated suggesting a key role in aging for the so-called nutrient-sensitive signaling pathways: the insulin/IGF-1 pathway, the AMP kinase pathway (AMPK), the mTOR kinase pathway, and the sirtuin pathway (López-Otín et al., 2016; Riera et al., 2016). These pathways keep complex interactions among them at several levels, and one of them is Ca^2+^ signaling, a key messenger in cellular activation.

Both the mTOR and AMPK cascades are related to Ca^2+^ signaling, but the specific mechanisms are not known in detail (Decuypere et al., 2013). First, AMPK can be phosphorylated and activated by Ca^2+^-calmodulin protein kinase β (CaMKKβ or CaMKK2). In turn, AMPK inhibits mTORC1, so that this pathway provides an indirect link between the Ca^2+^ signal and mTOR. According to this mechanism, cytosolic [Ca^2+^] ([Ca^2+^]_c_) increase would inhibit mTOR, thus activating processes such as autophagy. In fact, there is a large body of evidence showing that [Ca^2+^]_c_ signals from different sources can trigger autophagy by this “canonical” CaMKKβ-AMPK-mTORC1 pathway (Høyer-Hansen et al., 2007; Cárdenas and Foskett, 2012; Bootman et al., 2018).

On the other hand, there is also evidence that Ca^2+^ could have the contrary effect, that is, a direct activation of mTORC1. In this alternative pathway, [Ca^2+^]_c_ signals would have an inhibitory effect on autophagy. For example, addition of amino acids to nutrient-deficient cells activates mTORC1, leading to suppression of autophagy (Carroll et al., 2015). It has been reported that this effect can be mediated by an amino acid-induced increase in [Ca^2+^]_c_ that activates mTORC1 through binding of the Ca^2+^-calmodulin complex to a kinase (PI3K class III) necessary for the activation of mTOR (Gulati et al., 2008). Similarly, it has been reported that physical activity generates muscle hypertrophy through activation of Ca^2+^ entry into the cytosol, which would induce a direct activation of mTOR, responsible for initiating the hypertrophy process (Ito et al., 2013). In turn, it has also been described that mTOR activates the store-operated Ca^2+^-entry pathway (SOCE), through an increase in the expression of the STIM1/Orai1 proteins responsible for this pathway (Ogawa et al., 2012).

One of the key points in Ca^2+^ signaling linked to the activity of mTOR and AMPK is the inositol trisphosphate receptor (IP_3_R), which releases Ca^2+^ from the endoplasmic/sarcoplasmic reticulum (ER/SR) and is able to transfer Ca^2+^ from the ER to the mitochondria through close contacts that connect both organelles. These contacts, called Mitochondrial Associated ER Membranes (MAMs), contain both IP_3_R and mitochondrial Ca^2+^ uniporters (MCU), the main channel responsible for mitochondrial Ca^2+^ uptake. In addition, it has been reported that mTORC2 is also an important component of MAMs, able to activate AKT kinase, which may then phosphorylate and inhibit IP_3_R (Betz et al., 2013). Moreover, mTOR is also able to directly phosphorylate and activate IP_3_R (MacMillan et al., 2005; Frégeau et al., 2011; Régimbald-Dumas et al., 2011).

In turn, IP_3_R activity can also modulate the nutrient sensitive pathways. When ER Ca^2+^-release mediated by IP_3_R is reduced, both Ca^2+^-entry and ATP synthesis in mitochondria decrease. The resulting energy depletion activates AMPK, which then activates autophagy. The activity of the IP_3_R and the Ca^2+^-transfer between ER and mitochondria may therefore control AMPK and autophagy, and the final outcome may depend on the [Ca^2+^] transients induced in both cytosol and mitochondria after IP_3_R activation. ER-Ca^2+^ transfer to mitochondria fuels ATP production and decreases AMPK activity and autophagy. ER-Ca^2+^ release to the cytosol may activate AMPK and autophagy via CAMKKβ or alternatively activate directly mTOR and suppress autophagy (Cárdenas and Foskett, 2012; Parys et al., 2012; Bootman et al., 2018). Finally, resveratrol treatment decreases ER calcium storage and store-operated Ca^2+^-entry, inducing ER stress, activating AMPK and inhibiting the mTOR pathway (Selvaraj et al., 2016).

In conclusion, there is little doubt that [Ca^2+^]_c_ dynamics modulates mTOR, AMPK and autophagy in a number of ways, although a complete understanding of the mechanisms involved requires much further work. We believe therefore that Ca^2+^ signaling is a route that is worth exploring for the modulation of nutrient-sensitive pathways and aging. In particular, the level of [Ca^2+^] in the ER ([Ca^2+^]_ER_) appears to be closely related to the activity of the nutrient sensitive pathways. In this work we have studied the effect of the inhibition of the sarco-endoplasmic reticulum Ca^2+^ ATPase (SERCA) on survival in *C. elegans*. The rationale of this treatment is that submaximal SERCA inhibition should decrease [Ca^2+^]_ER_, leading to a reduced Ca^2+^ release from the ER and a smaller Ca^2+^ transfer from ER to mitochondria. This effect should in theory activate AMPK, inhibit mTOR and have a pro-longevity effect. Our data show in fact that SERCA inhibitors induced a significant increase in *C. elegans* lifespan, suggesting that Ca^2+^ homeostasis plays an important role in the mechanisms responsible for aging and survival.

## Materials and Methods

### [Ca^2+^]_ER_ Measurements With Aequorin in HeLa Cells

HeLa cells expressing double-mutated aequorin targeted to the ER (de la Fuente et al., 2013) were reconstituted with coelenterazine i, placed in the perfusion chamber of a purpose-built luminometer and perfused with external medium prior to the stimuli.

### *C. elegans* Strains and Maintenance

Strains used were: AQ2038, integrated strain expressing cytosolic cameleon 2.1. (YC2.1) on pharynx due to the promoter sequence of the myo-2 gene (*pmyo-2::YC2.1*) (Alvarez-Illera et al., 2016), kindly provided by Drs. Robyn Branicky and W. Schafer, MRC Laboratory of Molecular Biology, Cambridge, United Kingdom. Its lifespan was not significantly different from that of the N2 strain (data not shown), and it is used here as a control strain to study the effects of the SERCA inhibitors. The mutant *eat-2* strain (ad1113) was obtained from the Caenorhabditis Genetics Center. Worms were maintained and handled as previously described (Stiernagle, 2006). Hardened agar was seeded with *Escherichia coli* (OP50) and all the strains were maintained at 20°C.

### Preparation of the γ-Cyclodextrin Inclusion Compounds

The γ-cyclodextrin inclusion compounds were prepared as described before (Kashima et al., 2012). Briefly, a 230 mg/ml water solution of γ-cyclodextrin was mixed 10:1 with a 50 mM DMSO solution of the corresponding compound, stirred in shaker at 1200 rpm during 20 h and centrifuged at 12,500 rpm for 10 min. The supernatant was carefully discarded and the resulting inclusion compound was dried in the hood and dissolved in M9 buffer. The inclusion compounds containing either 2,5-BHQ, 2,6-BHQ or thapsigargin were added directly to the plates in the amounts indicated before transferring the worms for the lifespan assay. In the case of 2,5-BHQ, in some assays it was dissolved directly in the Nematode Growth Medium (NGM) agar by strong stirring at the concentrations indicated. In the case of thapsigargin, in some assays it was added directly to the plate. 10 μl of either 10 or 50 μM thapsigargin were added on top of the 10 ml OP50-seeded NGM agar to obtain final concentrations (assuming homogeneous distribution) of 10 and 50 nM, respectively.

### *C. elegans* Lifespan Assay

Eggs were obtained as described previously (Stiernagle, 2006) and transferred to *E. coli* (OP50) seeded NGM plates, either control plates or plates prepared in the presence of the required drug. For each assay, around 100 synchronized young adults (day 1) were transferred to *E. coli* (OP50) seeded NGM plates (35 mm plates, 10 worms/plate) containing 15 μM Fluorodeoxyuridine (FUdR) to avoid progeny. Control and drug-containing assays were always carried out in parallel. Plates were scored for dead worms every day. Worms that did not respond to touch with a platinum wire were considered dead. Age refers to days following adulthood. Plates with fungal contamination during the first 10 days of the assay were excluded from the study. Missing worms, individuals with extruded gonad or desiccated by crawling in the edge of the plate were censored, as well as plates with fungal contamination after the first 10 days. Control and drug-containing plates were kept close together in a temperature-controlled incubator set at 20°C. Statistics was made with the SPSS software using the Kaplan-Meier estimator and the log-rank routine for significance.

### Materials

2,5-BHQ and 2,6-BHQ were form Sigma-Aldrich, Madrid, Spain. Thapsigargin was from Abcam, Madrid, Spain. γ-cyclodextrin was from PanReac, Barcelona, Spain. FuDR was from Alfa Aesar, Karlsruhe, Germany. Other reagents were from Sigma, Madrid, Spain or Merck, Darmstadt, Germany.

## Results

### The SERCA Inhibitor 2,5-BHQ Extends *C. elegans* Lifespan More Than the Inactive Analog 2,6-BHQ

In mammalians there are three SERCA isoforms, encoded by the ATP2A 1-3 genes, and further isoform diversity is generated by alternative processing of the primary gene transcripts at a conserved 3*^prime^*-terminal site (Lytton et al., 1992; Vandecaetsbeek et al., 2011). In the nematode *Caenorhabditis elegans*, there is only one SERCA homolog, encoded by the *sca-1* gene (Cho et al., 2000; Zwaal et al., 2001), which has about 70% amino acid identity and 80% similarity to the three human SERCA proteins (Cho et al., 2000). The *sca-1* transcript is also alternatively spliced in a way similar to that of mammalian SERCA2 gene, generating two protein variants which show differential functional characteristics and expression patterns (Cho et al., 2000). As all the mammalian SERCA isoforms, the *C. elegans* protein is also inhibited by thapsigargin, a very potent and specific irreversible inhibitor of SERCA pumps (Zwaal et al., 2001). In addition, all the known SERCA pumps are also reversibly inhibited by the small compound 2,5-di-tert-butylhydroquinone (2,5-BHQ). We have therefore used here both 2,5-BHQ and thapsigargin to study their effect on worm lifespan.

**FIGURE 1:**
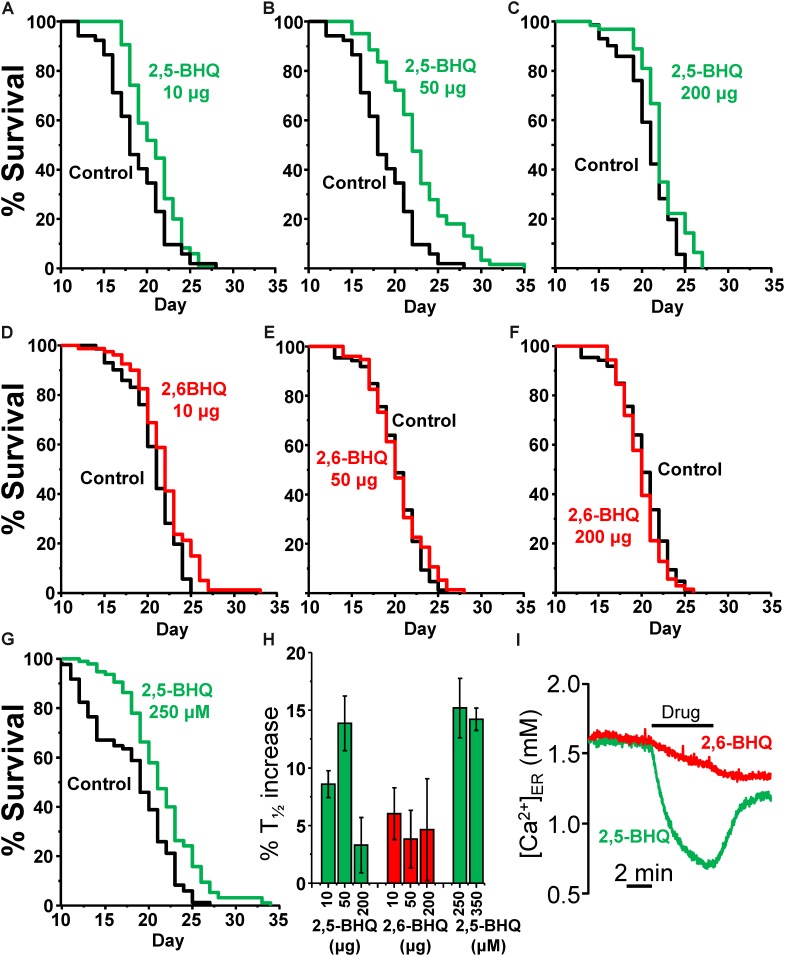
Effects of 2,5-BHQ and 2,6-BHQ on survival in *C. elegans*. Panels A–F show representative survival plots corresponding to parallel lifespan assays performed using γ-cyclodextrin-inclusion compounds in the following conditions: Control/2,5-BHQ (**A**:10 μg; **B**:50 μg; **C**:200 μg) and Control/2,6-BHQ (**D**:10 μg; **E**:50 μg; **F**:200 μg), respectively. **(G)** shows a representative survival plot from a Control/2,5-BHQ lifespan assay in which 250 μM 2,5-BHQ was dissolved in the NGM agar. The assays shown correspond to those marked in bold in **Table 1**. **(H)** shows the mean increase in survival obtained in a series of lifespan assays of each kind as those shown in the figure (more details of each assay in **Table 1**). **(I)** shows the effect of 10 μM of either 2,5-BHQ or 2,6-BHQ on the [Ca^2+^]_ER_ level in HeLa cells. This experiment is representative of 6-7 similar ones.

As a control for the activity of 2,5-BHQ, we have used 2,6-BHQ, a compound which is inactive on the SERCA (see **Figure 1I**), but should have very similar side effects, such as, for example, antioxidant properties. **Figure 1I** shows the effects of either 2,5-BHQ or 2,6-BHQ on the [Ca^2+^]_ER_ measured in HeLa cells expressing ER-targeted aequorin. Addition of 2,5-BHQ rapidly induced a decrease in [Ca^2+^]_ER_ due to the inhibition of the SERCA pump. In contrast, addition of 2,6-BHQ hardly produced any effect.

Because of the poor water solubility of 2,5-BHQ, we decided to use inclusion compounds for drug administration to the worms. 2,5-BHQ was enclosed in a chemical cover of γ-cyclodextrin, forming an inclusion compound (Kashima et al., 2012), which was then added to the plates together with the *E. coli* OP50. Young adults (day 1) were then transferred to these plates to start the lifespan assay. In this way, worms ingest the inclusion compound by the pharynx together with the food, and the active 2,5-BHQ is then released directly into the digestive tube. **Table 1** shows the results of a series of lifespan assays performed with three concentrations of either 2,5-BHQ or 2,6-BHQ. **Figure 1** shows representative lifespan assays performed in each of the conditions (2,5-BHQ, panels A–C; 2,6-BHQ, panels D–F). Although 2,6-BHQ produced small increases in survival, the pro-survival effect was much larger in the case of 2,5-BHQ, and its effect was concentration-dependent. The effect was maximum when 50 μg of the inclusion compound was added to the plate, and for this concentration all the lifespan assays were highly significant and produced increases in survival between 8.2 and 21.3% (mean 13.9 ± 2.4%). The effects on lifespan were smaller at concentrations of 2,5-BHQ below and above 50 μg. In contrast, for 2,6-BHQ, all the concentrations used produced smaller and similar increases in survival, and the effect showed a much higher variability.

**Table 1:** Lifespan assays performed with 2,5-BHQ and 2,6-BHQ in wild-type worms.

DRUG	T_½_ Drug (days)	N Drug	T_½_ Control (days)	N Control	% T_½_ increase	*p*-value Drug vs. Control	Mean % T_½_ increase
2,5-BHQ 10 μg	18.5	76/97	17.0	69/81	8.9	< 0.0001	**8.6 ± 1.2**
	22.2	45/60	20.1	52/64	10.5	< 0.018	
	21.5	77/105	20.4	86/109	5.3	< 0.006	
	22.4	73/100	21.0	71/95	6.6	< 0.0001	
	**21.1**	**85/106**	**18.9**	**52/62**	**11.6**	**< 0.001**	
2,5-BHQ 50 μg	18.4	69/82	17.0	69/81	8.2	< 0.0001	**13.9 ± 2.4**
	23.5	42/60	20.1	52/64	17.2	< 0.0001	
	22.6	63/80	20.4	86/109	10.6	< 0.0001	
	23.6	81/98	21.0	71/95	12.0	< 0.0001	
	**23.0**	**61/80**	**18.9**	**52/62**	**21.3**	**< 0.0001**	
2,5-BHQ 200 μg	16.6	77/101	17.0	69/81	-2.3	0.193	**3.3 ± 2.4**
	21.3	63/82	20.1	52/64	6.2	0.099	
	19.9	60/82	20.4	86/109	-2.5	0.171	
	**22.3**	**63/80**	**21.0**	**71/95**	**5.9**	**< 0.005**	
	20.7	59/80	18.9	52/62	9.3	< 0.015	
2,6-BHQ 10 μg	17.7	62/80	17.0	69/81	4.1	0.046	**6.0 ± 2.3**
	22.5	63/84	20.1	52/64	11.9	< 0.001	
	20.2	78/101	20.4	86/109	-0.9	0.421	
	**22.1**	**80/99**	**21.0**	**71/95**	**5.2**	**< 0.006**	
	20.8	71/85	18.9	52/62	9.7	< 0.01	
2,6-BHQ 50 μg	18.3	68/84	17.0	69/81	7.6	< 0.0001	**3.8 ± 2.5**
	18.4	53/60	18.9	52/64	-2.3	0.265	
	**20.8**	**75/102**	**20.4**	**86/109**	**1.8**	** 0.289**	
	20.5	57/81	18.9	52/64	8.2	< 0.022	
2,6-BHQ 200 μg	19.4	45/59	17.0	69/81	14.0	< 0.0001	**4.6 ± 4.4**
	18.1	53/60	20.1	52/64	-10.0	< 0.001	
	**20.2**	**71/98**	**20.4**	**86/109**	-**1.0**	**0.314**	
	23.1	53/64	21.0	71/95	9.6	< 0.0001	
	20.9	59/83	18.9	52/62	10.5	< 0.007	
2,5-BHQ 250 μM	25.3	85/97	22.6	82/100	11.9	< 0.0001	**15.2 ± 2.6**
	**21.6**	**95/105**	**18.5**	**85/103**	**16.6**	**< 0.0001**	
	20.5	96/108	18.5	82/90	10.4	< 0.006	
	22.4	80/94	18.4	74/80	21.8	< 0.0001	
2,5-BHQ 350 μM	25.9	96/111	22.6	82/100	14.6	< 0.0001	**14.2 ± 1.0**
	20.8	99/106	18.5	85/103	12.4	< 0.002	
	21.0	96/109	18.5	82/90	13.1	< 0.001	
	21.5	71/84	18.4	74/80	16.8	< 0.0001	


*The table shows the drug concentration used in each series of assays, the half-life (T_½_) of the worms incubated with the drug obtained from the Kaplan-Meier analysis, the number of worms in the drug-containing assay (final/total), the half-life (_½_) of the control worms, the number of worms in the control assay (final/total), the % increase in the half-life, the significance of the difference obtained from the log-rank test and the mean ± s.e. increase in half-life from all the series made with the same drug concentration. Concentrations in μg for γ-cyclodextrin-inclusion compounds and in μM for compound dissolved in NGM agar. In bold, series shown in the survival plots of **Figure 1**.*

In spite of the low water solubility of BHQ, we have also tested the effect of two concentrations of 2,5-BHQ directly dissolved in the NGM agar after strong stirring, 250 and 350 μM. Both produced also an increase in the *C. elegans* lifespan of similar magnitude as that induced by 50 μg of the compound (**Table 1** and **Figure 1G**) showing that the effect is not dependent on the method of administration of the drug. Panel H shows a comparison of the mean increase in survival obtained in every condition.

### The Specific SERCA Inhibitor Thapsigargin Extends *C. elegans* Lifespan at Low Concentrations

We have then studied the effect of thapsigargin, a very potent and specific irreversible inhibitor of SERCA pumps, on the lifespan of the worms. For drug administration, we have used two different mechanisms, either inclusion compounds or direct addition to the plates. In the first method, the inclusion compound with thapsigargin was prepared and used as mentioned above. In the second method, as previously described (Zwaal et al., 2001), the drug was added directly to the plates seeded with *E. coli* (OP50), and young adults (day 1) were then transferred to these plates to start the lifespan assay.

**FIGURE 2:**
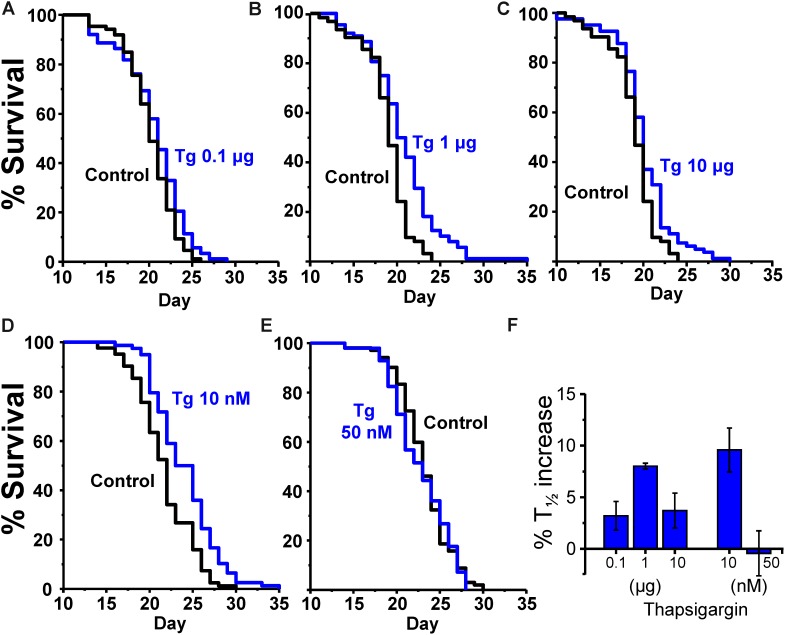
Effects of thapsigargin on survival in *C. elegans*. **(A–C)** show representative survival plots corresponding to parallel lifespan assays performed using γ-cyclodextrin-inclusion compounds in the following conditions: Control/thapsigargin (**A**:0.1 μg; **B**: 1μg; **C**:10 μg). **(D,E)** Show representative survival plots from Control/thapsigargin lifespan assays in which thapsigargin was directly added onto the OP50-seeded NGM agar (final concentration 10 or 50 nM). The assays correspond to those marked in bold in **Table 2**. **(F)** Shows the mean increase in survival obtained in a series of lifespan assays of each kind as those shown in the figure (more details of each assay in **Table 2**).

The same effects were obtained with both drug administration methods. **Table 2** shows the results of a series of lifespan assays performed with three concentrations of γ-cyclodextrin enclosed thapsigargin (0.1, 1, and 10 μg) and two concentrations of thapsigargin added directly to the plates (10 and 50 nM). Representative lifespan assays performed in each of the conditions are shown in **Figures 2A–E**, together with the mean increase in survival obtained from the different assays made at each thapsigargin concentration (**Figure 2F**). Thapsigargin produced also an increase in survival which was dependent on the concentration. The effect was maximum at 1 μg of the inclusion compound (mean increase 8.0 ± 0.3%), and was smaller at concentrations below and above that. Also, when the compound was added directly to the plates, the increase in lifespan was obtained at 10 nM (mean increase 9.6 ± 2.1%), but disappeared at 50 nM.

**Table 2:** Lifespan assays performed with thapsigargin in wild-type worms.

DRUG	T_½_ Drug (days)	N Drug	T_½_ Control (days)	N Control	% T_½_ increase	*p*-value Drug vs. Control	Mean % T_½_ increase
Thapsigargin 0.1 μg	17.0	80/96	17.0	69/81	0.2	0.89	**3.2 ± 1.4**
	19.9	69/82	19.3	62/80	3.2	0.107	
	21.4	61/82	20.1	52/64	6.9	0.057	
	**20.9**	**88/106**	**20.4**	**86/109**	**2.6**	**< 0.038**	
Thapsigargin 1 μg	**21.0**	**88/102**	**19.3**	**62/80**	**8.8**	**< 0.0001**	**8.0 ± 0.3**
	21.5	70/80	20.1	52/64	7.4	< 0.028	
	18.3	80/93	17.0	69/81	7.8	< 0.0001	
	22.1	60/81	20.4	86/109	8.1	< 0.004	
Thapsigargin 10 μg	18.2	89/102	17.0	69/81	7.0	< 0.0001	**3.7 ± 1.7**
	**20.4**	**81/99**	**19.3**	**62/80**	**5.6**	**< 0.014**	
	20.6	79/101	20.1	52/64	2.8	0.37	
	20.3	90/109	20.4	86/109	-0.6	0.974	
Thapsigargin 10 nM	**24.2**	**78/103**	**21.9**	**82/101**	**10.6**	**< 0.0001**	**9.6 ± 2.1**
	21.5	50/60	21.0	71/95	2.1	0.207	
	23.4	53/82	21.7	70/102	8.1	< 0.005	
	25.2	96/108	22.2	102/115	13.5	< 0.0001	
	25.7	67/80	22.6	73/84	13.6	< 0.0001	
Thapsigargin 50 nM	20.6	86/103	21.9	82/101	-6.0	< 0.002	-**0.44 ± 2.2**
	22.0	70/100	21.7	70/102	1.7	0.927	
	**22.9**	**97/107**	**23.2**	**102/115**	-**1.7**	** 0.616**	
	23.6	85/101	22.6	73/84	4.2	0.15	


*Concentrations in μg for γ-cyclodextrin-inclusion compounds and in nM for thapsigargin added directly over the OP50-seeded NGM agar. In bold, series shown in the survival plots of **Figure 2**. Other details as in **Table 1**.*

### SERCA Inhibitors Extend the Lifespan in *Eat-2* Mutants

**FIGURE 3:**
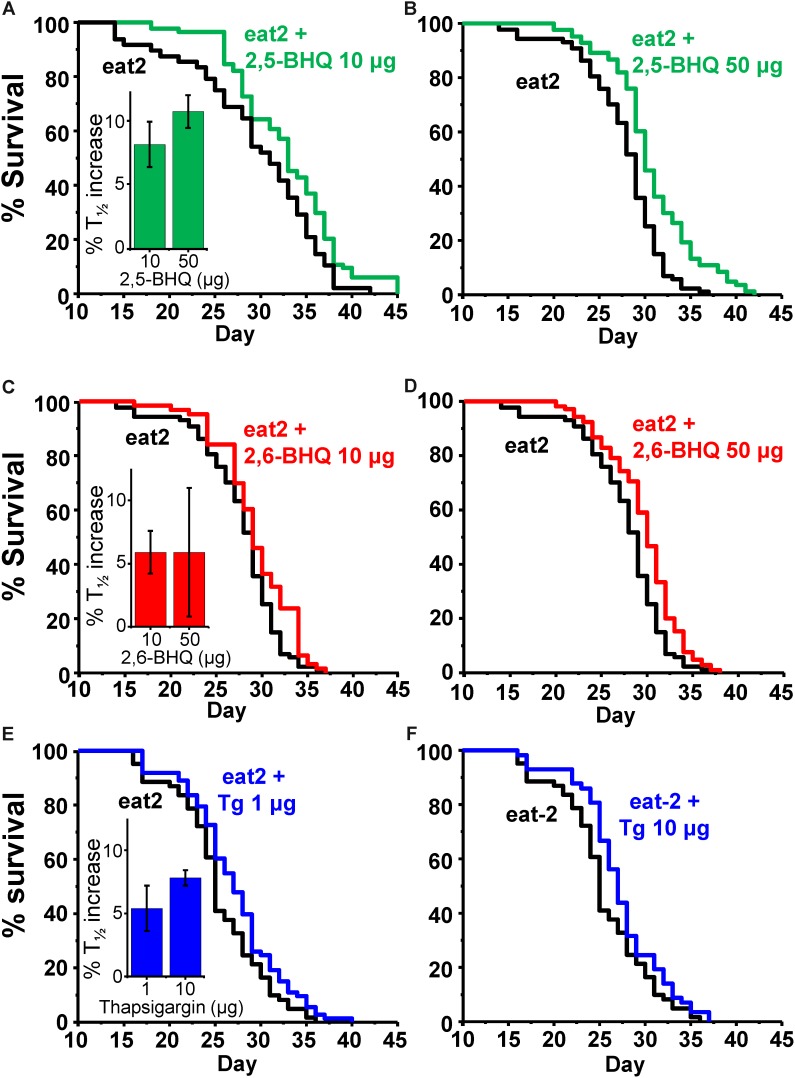
Effect of SERCA inhibitors on survival in *eat-2 C. elegans* mutants. The panels show representative survival plots corresponding to parallel lifespan assays performed using γ-cyclodextrin-inclusion compounds in the following conditions: *eat-2/ eat-2* + 2,5-BHQ (**A**:10 μg; **B**:50 μg), *eat-2/ eat-2* + 2,6-BHQ (**C**:10 μg; **D**:50 μg) and *eat-2/ eat-2* + thapsigargin (**E**:1 μg, **F**:10 μg). The assays correspond to those marked in bold in **Table 3**. The inserts show the mean increase in survival obtained in a series of lifespan assays of each kind as those shown in the figure (more details of each assay in **Table 3**).

To exclude that the mechanism of the increased lifespan in the presence of the SERCA inhibitors could be related to caloric restriction, we have studied the effects of the SERCA inhibitors on the survival of *eat-2* mutants. These mutants have a reduced rate of pharynx pumping and an increased lifespan, which has been shown to be due to caloric restriction (Lakowski and Hekimi, 1998). **Table 3** shows the results of a series of lifespan assays performed with several concentrations of 2,5-BHQ, 2,6-BHQ and thapsigargin in *eat-2 (ad1113)* mutants. Representative plots of assays made at each of the conditions studied are shown in **Figure 3**. The results show that SERCA inhibitors increased also the lifespan of *eat-2 (ad1113)* worms with a similar magnitude to that found in wild-type worms. Therefore, this suggests that caloric restriction is not involved in the mechanism of the lifespan increase induced by these compounds.

**Table 3:** Lifespan assays performed with the SERCA inhibitors in *eat-2* mutants.

DRUG	T_½_ Drug (days)	N Drug	T_½_ Control (days)	N Control	% T_½_ increase	*p*-value Drug vs. Control	Mean % T_½_ increase
2,5-BHQ 10 μg	**33.1**	**84/102**	**30.2**	**48/60**	**9.7**	**< 0.035**	**8.3 ± 1.8**
	29.2	59/71	27.9	87/96	4.8	< 0.038	
	28.2	50/61	25.6	61/65	10.4	< 0.010	
2,5-BHQ 50 μg	32.7	77/103	30.2	48/60	8.3	0.129	**10.9 ± 1.3**
	**31.0**	**83/96**	**27.9**	**87/96**	**11.3**	**< 0.0001**	
	28.9	61/67	25.6	61/65	12.9	< 0.001	
2,6-BHQ 10 μg	31.4	56/78	30.2	48/60	3.9	0.9	**5.9 ± 1.7**
	**29.2**	**63/68**	**27.9**	**87/96**	**4.7**	**< 0.044**	
	28.0	71/87	25.6	61/65	9.2	< 0.006	
2,6-BHQ 50 μg	29.3	26/104	30.2	48/60	-3.1	0.153	**5.9 ± 5.1**
	**29.7**	**105/116**	**27.9**	**87/96**	**6.4**	**< 0.002**	
	29.3	35/43	25.6	61/65	14.4	< 0.0001	
Thapsigargin 1 μg	28.4	56/68	27.9	87/96	1.8	0.9	**5.4 ± 1.8**
	**27.4**	**73/83**	**25.6**	**61/65**	**7.1**	**< 0.028**	
	23.4	57/85	21.4	64/95	9.3	< 0.001	
Thapsigargin 10 μg	**27.4**	**57/63**	**25.6**	**61/65**	**7.2**	**< 0.039**	**7.8 ± 0.6**
	23.2	70/101	21.4	64/95	8.4	< 0.007	


*In bold, series shown in the survival plots of **Figure 3**. Other details as in **Table 1**.*

## Discussion

Intracellular Ca^2+^ dynamics is a key controller of cellular metabolism. The rate of mitochondrial ATP synthesis depends on the [Ca^2+^] inside mitochondria ([Ca^2+^]_M_), which stimulates several key dehydrogenases responsible for NADH production (McCormack et al., 1990). As a consequence of this, an increase in [Ca^2+^]_M_ immediately leads to an increase in ATP production (Robb-Gaspers et al., 1998; Jouaville et al., 1999). The increase in [Ca^2+^]_M_ is generally a consequence of the stimulation of Ca^2+^-release from the ER that occurs during cell activation. This mechanism constitutes a basic homeostatic response that associates cell activation to energy production, in order to cover the energy requirements of the response to the stimulus.

On the other hand, the increase in the level of [Ca^2+^]_c_ is directly responsible for the activation of many cellular responses that consume energy, such as muscle contraction, secretion, proliferation, hypertrophy and many metabolic reactions (Berridge et al., 2000). In addition, many Ca^2+^ signals adopt prolonged oscillatory patterns that require a continuous activity of the Ca^2+^ pumps to restore the Ca^2+^ gradients after each oscillation. These oscillatory Ca^2+^ patterns consume large amounts of energy only at the level of the Ca^2+^ pumps, both in the plasma membrane and in the ER. It has been shown, for example, that ATP consumption by SERCA pumps accounts for 40–50% of the resting metabolic consumption in mouse skeletal muscle (Smith et al., 2013).

The concepts of energy balance and nutrient-sensitive pathways are considered nowadays key determinants of lifespan (López-Otín et al., 2016; Riera et al., 2016). As Ca^2+^ signaling is tightly related to both of them, we decided to study the effects of Ca^2+^ modulators on survival. On the first place, we have investigated the effect of submaximal inhibition of the SERCA pumps, the Ca^2+^ pumps responsible for Ca^2+^-storage in the ER. The level of [Ca^2+^]_ER_ is key to determine the amount of Ca^2+^ released during cell stimulation, not only because the rate of Ca^2+^ release depends on the Ca^2+^-gradient between the ER and the cytosol, but mainly because [Ca^2+^]_ER_ is a very important activator of both IP_3_R and ryanodine receptors (Yamasaki-Mann et al., 2010; Zhang et al., 2015). As a consequence, partial Ca^2+^ depletion of the ER should reduce both [Ca^2+^]_c_ signaling and ER-mitochondria Ca^2+^ transfer during cell stimulation.

To test this hypothesis, we have used two inhibitors of SERCA pumps, 2,5-BHQ and thapsigargin. Thapsigargin is the most potent and specific SERCA inhibitor available. It binds SERCA with a Kd in the sub-nM range (Thastrup et al., 1990) and stabilizes the Ca^2+^-free conformation, a property which has been useful to obtain SERCA crystals for diffraction studies (Toyoshima et al., 2000; Inesi et al., 2005, 2006). Thapsigargin has been reported to act also on different targets, particularly on plasma membrane Ca^2+^ channels (Rossier et al., 1993; Nelson et al., 1994; Shmigol et al., 1995). However, this off-target effect required always much higher concentrations, in the micromolar range. It is therefore considered highly specific for SERCA inhibition at low concentrations, in the nM range.

Regarding 2,5-BHQ, it has been known to be a SERCA inhibitor for more than 30 years (Moore et al., 1987). It binds SERCA close to the thapsigargin-binding site, although not in the same place, and stabilizes also the Ca^2+^-free conformation (Inesi et al., 2005). The Kd for SERCA inhibition by 2,5-BHQ is higher, around 1–2 μM (Moore et al., 1987; Llopis et al., 1991), and this compound has been shown to inhibit also some additional targets, including L-type Ca^2+^ channels (Nelson et al., 1994; Scamps et al., 2000; Miller et al., 2015) other types of Ca^2+^ channels (Scamps et al., 2000) or the plasma membrane Ca^2+^ ATPase (Luo et al., 2000). Most of these off-target effects of 2,5-BHQ required also higher concentrations of the drug (IC50 ≥ 30 μM), but could be significant at the concentrations usually applied to obtain maximum SERCA inhibition (∼10 μM). In addition, 2,5-BHQ has also been shown to generate superoxide anion (Tsukii et al., 1996) and inhibition of L-type Ca^2+^ channels by this compound (with IC_50_ = 66 μM) has been attributed to this effect (Fusi et al., 2001). On the other hand, this compound belongs to a family of phenolic antioxidants, some of which are used in food preservation. To account for these alternative effects of 2,5-BHQ, we have used the analog 2,6-BHQ, which is inactive on the SERCA pump but should have similar chemical properties, particularly on oxidative processes. Regarding the 2,5-BHQ concentration in our experiments, it is difficult to know the exact concentration inside the worms, as it depends on the lipophilicity and bioavailability of the drug. Usually, the drug concentrations in the NGM agar required to produce effects in the worms are 10–100 fold higher than those necessary in cell cultures. Assuming that we are producing submaximal inhibition of SERCA, worm concentrations should be close to the IC_50_, around 1–2μM.

Our data show in fact that submaximal inhibition of SERCA pumps with 2,5-BHQ or thapsigargin produced a significant increase in the lifespan in *C. elegans* worms. The effect on survival of both inhibitors was concentration-dependent, reached a maximum at a particular concentration, and decayed below and above it. Our data show that, although 2,6-BHQ produced a small increase in survival, the lifespan extension induced by 2,5-BHQ was 3-fold larger, suggesting that SERCA inhibition is clearly involved in the effect. The effect of 2,6-BHQ could be attributed to its antioxidant properties, although antioxidants may have variable effects of *C. elegans* lifespan (Desjardins et al., 2017). Regarding thapsigargin, it is a much more potent and specific SERCA inhibitor, so that much lower concentrations need to be used to obtain the effects and fewer side-effects are expected to occur. It is also an irreversible inhibitor of SERCA, and it has been shown before that inhibits the SERCA pump in *C. elegans* and that concentrations in the plate above ∼200 nM are deleterious for worm fertility and growth (Zwaal et al., 2001). This is consistent with the fact that knockout of the SERCA homolog in C. elegans, *sca-1*, results in embryonic and larval lethality (Cho et al., 2000; Zwaal et al., 2001). In our hands, an increase in the lifespan was obtained with a much lower concentration, just 10 nM, and the effect disappeared when the concentration was increased to 50 nM. At this concentration, the effects of thapsigargin should be highly specific on SERCA.

The molecular mechanisms linking SERCA inhibition and lifespan extension still await clarification. We have seen that the SERCA pump inhibitors increased also with a similar magnitude the lifespan of *eat-2 (ad1113)* mutant worms, a model for caloric restriction because of the reduced rate of pharynx pumping (Lakowski and Hekimi, 1998). This suggests that the increase in lifespan induced by the compounds is not due to caloric restriction, but rather to the effect of SERCA inhibition on Ca^2+^ signaling. The SERCA pump plays a key role in shaping the intracellular [Ca^2+^] transients. Its activity determines the level of filling with Ca^2+^ of the ER/SR, which is one of the factors conditioning the rate of Ca^2+^ release through either IP_3_R or ryanodine receptors. In addition, ER/SR Ca^2+^ reuptake after stimulation also depends on SERCA activity. Our hypothesis is that a decrease in SERCA activity would reduce [Ca^2+^]_ER_, with the subsequent decrease in Ca^2+^ release, Ca^2+^ transfer from ER to mitochondria and metabolic activity. It is remarkable that SERCA is one of the proteins that undergo a largest decrease in concentration during aging in *C. elegans*. In fact, *sca-1* decreases in abundance nearly 10-fold from day 4 to day 10 of worm life (Copes et al., 2015). This phenomenon is not specific of *C. elegans*, because a marked decrease in SERCA2a mRNA and activity during aging has also been reported in mammals (Feridooni et al., 2015). This decrease could be interpreted in several ways, but perhaps could reflect the fact that excess Ca^2+^ signaling is negative for survival during aging. Young individuals have a higher SERCA content, and this may be optimal for rapid response to external stimuli. Older individuals have instead a much smaller SERCA content, and this may dampen Ca^2+^ signaling and favor survival. Submaximal SERCA inhibition may extend lifespan in this way. Further work will be necessary to clarify the molecular mechanism involved in the effect of SERCA inhibitors. Measuring the changes in [Ca^2+^]_ER_ along the life of the worm, although challenging from a experimental point of view, would be a exciting approach. In addition, the decrease in [Ca^2+^]_ER_ should produce changes in the cytosolic Ca^2+^ dynamics, which could be detected in live treated or untreated worms. Using other mutants with alterations in nutrient-sensitive pathways or with ER stress may also provide clues on the mechanism of the pro-survival effect of SERCA inhibitors.

In summary, we have shown here that partial inhibition of SERCA pumps increases the lifespan in *C. elegans*. This indicates that Ca^2+^ signaling plays a role in the aging process and that reduction in [Ca^2+^]_ER_ and ER-Ca^2+^ release has a pro-longevity effect. Our data also suggest that Ca^2+^ signaling could be a promising novel target pathway to act on aging.

## Data Availability Statement

The raw data supporting the conclusions of this manuscript will be made available by the authors, without undue reservation, to any qualified researcher.

## Author Contributions

MM and JA designed the project. PG-C performed most of the lifespan experiments. JA-d-V and PA-I joined in performing some of them. JA wrote the manuscript and RF and MM helped in discussing and editing the manuscript. All authors read and approved the final manuscript.

## Conflict of Interest Statement

The authors declare that the research was conducted in the absence of any commercial or financial relationships that could be construed as a potential conflict of interest.
